# Lymphocyte activation markers predict the therapeutic response to immune checkpoint inhibitors: A case–control study

**DOI:** 10.1002/cam4.7418

**Published:** 2024-06-22

**Authors:** Takuya Iwamoto, Hajime Fujimoto, Tetsu Kobayashi, Esteban C. Gabazza

**Affiliations:** ^1^ Department of Pharmacy Mie University Hospital Tsu Japan; ^2^ Department of Pulmonary and Critical Care Medicine Mie University Graduate School of Medicine Tsu Japan; ^3^ Department of Immunology Mie University Graduate School of Medicine Tsu Japan

**Keywords:** biomarker, case–control study, immune checkpoint inhibitor, lymphocyte, non‐small cell lung cancer

## Abstract

**Background:**

Reports on changes in circulating immune cells induced by immune checkpoint inhibitors (ICIs) and their association with therapeutic responses are limited. This study aimed to analyze the interaction between ICIs and circulating blood cells in real‐world practice and identify biomarkers associated with therapeutic efficacy.

**Materials and Methods:**

Patients with non‐small cell lung cancer who received nivolumab, pembrolizumab, or atezolizumab monotherapy were eligible. Blood samples were collected before and 7–21, and 22–42 days after treatment, and changes in the number and characteristics of neutrophils and lymphocytes in the peripheral blood were analyzed. The efficacy of ICIs was evaluated based on the best overall response up to 3 months after treatment. The patients were divided into the following groups: patients with non‐progressive disease (non‐PD) and those with progressive disease (PD). Blood immunological factors related to treatment response were analyzed.

**Results:**

There were 16 and 27 patients in the non‐PD and PD groups, respectively. The non‐PD group had significantly lower pretreatment CD56^+^ cell (CD56^+^ IFN‐γ^+^ cell), and neutrophil counts at 7–21 days after ICI administration than the PD group. Additionally, there was a significant increase in CD8^+^ IFN‐γ^+^, and CD8^+^CD28^+^ IFN‐γ^+^ T‐cell counts 7–21 days after ICI administration in the non‐PD group. CD8^+^CD28^+^IFN‐γ^+^ T‐cell count greater than 94.6 cells/μL at 7–21 days after ICI administration was highly predictive with an AUC greater than 0.8 in the ROC curve analysis.

**Conclusions:**

Predictors of disease progression include a low activated CD56^+^ cell count, no increase in the neutrophil count before ICI therapy, and an increase of activated CD8^+^ cell count early after ICI administration. The findings will contribute to selecting patients with a favorable therapeutic response and early prediction of therapeutic response to ICI.

## Graphical abstract

Lymphocyte activation analysis of patients treated with immune checkpoint inhibitors.CD8+CD28+ IFN‐γ + T‐cell counts within 3 weeks of treatment may be useful in predicting drug response.

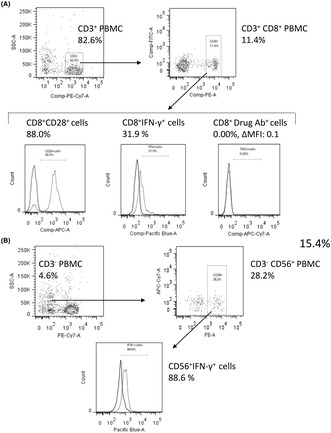

## 1 INTRODUCTION

Treatment with antibodies against programmed death‐ligand 1 (PD‐L1) or programmed cell death 1 protein (PD‐1), or immune checkpoint inhibitors (ICIs) induces unprecedented tumor responses in various cancer types.^1^ In a clinical trial comparing the efficacy of nivolumab, an ICI, with that of docetaxel as a second‐line treatment in patients with non‐small cell lung cancer (NSCLC), 8% of patients showed no progression at 1 year with docetaxel compared with 19% of patients who showed no progression with nivolumab, indicating that some patients continued to respond to treatment for a long period.^2^ However, only a limited number of patients can sustain the therapeutic effect over a long period. In addition, as ICIs are expensive, high medical costs become a problem with an increased number of patients using these drugs. Therefore, the characterization of tumor microenvironmental factors and patient immune function at baseline and early during treatment to identify patients who will respond to therapy is a research priority in cancer care.

PD‐L1 expression in tumors is a potential biomarker that has been investigated since the initiation of clinical trials with ICIs, and its correlation with the therapeutic efficacy of nivolumab and pembrolizumab for melanoma or NSCLC has been reported.^3^, ^4^, ^5^ Recently, PD‐L1‐expressing CD11b^+^ myeloid cells in the systemic circulation have also been reported to be associated with clinical response.^6^ However, a study in a mouse model in which *PD‐L1* was knocked out from tumor cells revealed no change in the anti‐tumor effect of the anti‐PD‐L1 antibody, indicating that PD‐L1 expression on tumors is not essential for PD‐1 to suppress T‐cell function.^7^ Moreover, since some patients with PD‐L1‐negative tumors show therapeutic responses, host immune function plays a major role in the therapeutic efficacy of ICIs.^8^, ^9^

A characteristic of cancer cells with a good response to ICIs is a high frequency of genetic mutations. Improved overall survival with cytotoxic T‐lymphocyte‐associated protein 4 (CTLA‐4) inhibitor in patients with melanoma harboring more than 100 mutations and improved median progression‐free survival in patients with lung cancer treated with pembrolizumab with a high mutation burden have been reported.^10^, ^11^ In clinical practice, nivolumab and pembrolizumab have been approved for solid tumors with a high frequency of microsatellite instability (MSI‐High).^12^, ^13^ A study using whole‐exome sequencing also revealed that 1782 somatic mutations on an average per tumor in mismatch repair‐deficient tumors, compared with 73 somatic mutations in mismatch repair‐proficient tumors, was associated with prolonged progression‐free survival.^14^ Additionally, it has been reported that tumor mutation burden (TMB) is significantly higher in responders than in non‐responders, and TMB > 23.1 Mut/Mb (TMB‐high) is associated with a survival benefit compared with TMB ≤23.1 Mut/Mb (TMB‐low or TMB‐intermediate) in patients with metastasized or unresectable melanoma.^15^

Regarding patient immune function, patients with high CD8^+^ T‐cell counts at invasive margins of the tumor,^16^ high peripheral blood lymphocyte counts, and low neutrophil counts^17^ have a better prognosis, and the intestinal microbiota influences the response to treatment.^18^, ^19^ CD8^+^ T‐cell signaling (TCR) induce PD‐1 expression in the cell surface, and when PD‐l binds to its ligands (PD‐L1 or PDL2), it inhibits TCR/CD28 signaling and T‐cell activation. Blockade of the PD‐1 pathway reinvigorates exhausted CD8^+^ T cells and can restore antitumor immune response.^20^ Furthermore, PD‐1, whose expression on CD8^+^ T cells increases following ICI administration, and CD28, a co‐stimulatory molecule that enhances T‐cell activity, are also attracting attention as candidate biomarkers for good clinical outcomes.^20^, ^21^ However, there are only a few reports on CD28 expression changes by ICIs and their association with therapeutic response. NK cells, known as CD56^+^ cells, are type of cytotoxic lymphocyte that is critical to the innate immune system and account for 5%–20% of all circulating lymphocytes in humans. The role of NK cells is analogous to that of cytotoxic T cells in the adaptive immune response, however, there has been little investigation on the contribution of NK cells to the therapeutic effects of ICI.

This exploratory prospective case–control study was conducted in patients with NSCLC with the hope of extending the hematological molecular markers that are expected to predict therapeutic response to ICI to clinical applications. The results will contribute significance to selecting patients with a favorable therapeutic response to ICI.

## 2 METHODS

### 2.1 Patient selection, sample collection, and measurements

This single‐center prospective study at Mie University Hospital involved patients with unresectable advanced or recurrent NSCLC at stage III or IV. The patients were 20 years or older, had a performance status (PS) of 0–1, and were scheduled to receive one of the following ICIs as a monotherapy: nivolumab (OPDIVO I.V. infusion; Ono Pharmaceutical Co., Ltd., Osaka, Japan), pembrolizumab (KEYTRUDA injection; Merck & Co., Inc., Rahway, NJ), or atezolizumab (TECENTRIQ for intravenous infusion; Chugai Pharmaceutical Co., Ltd., Tokyo, Japan). Patients were excluded if they required systemic treatment with steroids (prednisolone equivalent >10 mg/day) or other immunosuppressive agents within 14 days of enrollment or had concomitant autoimmune diseases. Cases in which treatment was discontinued within 2 weeks of ICI administration were also excluded. The PD‐L1 expression level was evaluated using the tumor proportion score, measured using PD‐L1 IHC 22C3 pharmDx, Dako (Agilent Technologies Japan, Ltd., Tokyo, Japan).

Residual blood samples from routine medical examinations were used in the assays. The samples were collected before ICI administration and 7–21 and 22–42 days after administration. Before the administration of ICIs, lymphocyte, neutrophil, CD3^−^ CD56^+^ cell, CD3^−^ CD56^+^ IFN‐γ^+^ cell, CD3^+^ CD8^+^ CD28^+^ T cell, and CD3^+^ CD8^+^ IFN‐γ^+^ T cell counts were measured. On Days 7–21 and 22–42, after treatment with ICIs, lymphocyte, neutrophil, CD3^−^ CD56^+^ cell, CD3^−^ CD56^+^ IFN‐γ^+^ cell, CD3^+^ CD8^+^ CD28^+^ T cell, and CD3^+^CD8^+^IFN‐γ^+^T cell counts, and drug binding to CD3^+^ CD8^+^ T cells were determined. The primary endpoint was non‐progression disease (non‐PD) for 3 months.

This study was approved by the Ethics Committee of Mie University (approval no. 3062), and written informed consent was obtained from each participant between November 2017 and August 2020. The target number of participants was set at 20 for each of the non‐PD and PD groups, with a ratio of 1:1 for each group and 10% would meet the exclusion criteria, for a total of 50 participants.

### 2.2 Assessment of activated T cells

To activate lymphocytes, PBMCs isolated from patient blood samples were incubated with phorbol 12‐myristate 13‐acetate and a calcium ionophore, A23187 (Sigma Chemicals, St. Louis, MO), for 5 h as described previously.^22^ Cell pellets were incubated for 15 min with PE‐Cy7‐labeled anti‐CD3 antibodies (Beckman Coulter, Tokyo, Japan); PE‐labeled anti‐CD4, anti‐CD8, or anti‐CD56 antibodies (BioLegend, Tokyo, Japan); and APC‐labeled anti‐CD28 (IL‐2R) antibodies (BioLegend). FITC‐labeled nivolumab, pembrolizumab, and atezolizumab were prepared using the Mix‐n‐Stain™ CFTM488A antibody labeling kit (FUJIFILM Wako Pure Chemical Corporation, Osaka, Japan), and FTIC‐labeled rituximab was also prepared using the kit and used as the isotype control. The cell pellets were treated with a cell permeation reagent for 20 min and reacted with Pacific blue‐labeled anti‐IFN‐γ antibodies (BioLegend). After rinsing, NK^+^ and CD8^+^ T cells were fractionated from CD3^−^ and CD56^+^ gates and CD3^+^ and CD8^+^ gates, respectively. The expression level of CD28^+^ cells in CD8^+^ T cells and IFN‐γ in CD8^+^ T cells was also measured. Flow cytometry was performed using a FACS Canto II flow cytometer (BD Biosciences) and FlowJo 7.6.5 software (BD Biosciences). Flow cytometric quantification of lymphocyte subsets was performed.

### 2.3 Pharmacodynamic analysis and statistical analysis

This study evaluated the pharmacodynamic changes of immune biomarkers in blood NK and CD8^+^ T cells and their association with response to therapy with ICs at 3 months (Response Evaluation Criteria in Solid Tumors criteria version 1.1). Patients were divided into PD and non‐PD groups 3 months after initiating ICIs. Hematological immune biomarkers were compared between the groups using the non‐parametric Mann–Whitney *U*‐test for numerical variables and Fisher's exact test for categorical variables. All tests of comparison between the two groups in this study showed the results of the exact *p*‐value, and the results of the approximate *p*‐value were not used. Receiver operating characteristic (ROC) curve analysis was performed to estimate the sensitivity, specificity, accuracy, and cutoff values of several hematological immune biomarkers and their association with the primary endpoint. Statistical analyses were performed using GraphPad Prism (version 9.3.1; GraphPad Software, San Diego, CA). Two‐sided *p*‐values were reported, and the results were considered significant at *p* < 0.05.

## 3 RESULTS

### 3.1 Enrolment of patients

**FIGURE 1:**
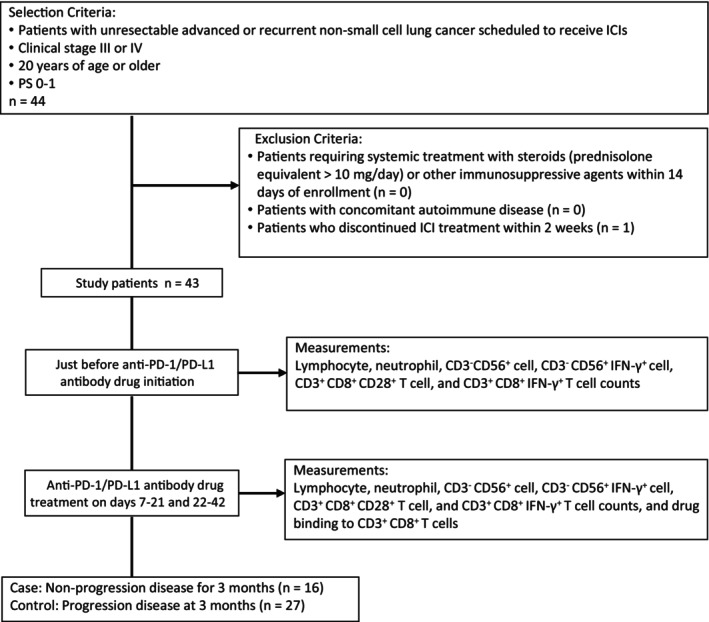
Study flow diagram and measurement items.

The study flow is presented in Figure 1. Forty‐four patients were enrolled in this study, and one patient discontinued ICI within 2 weeks after initiation. A total of 43 patients (29 men and 14 women) were eligible for analysis. The median age at entry was 71 years (youngest, 41 years; oldest, 84 years). The patients' demographics of PD (*n* = 27) or non‐PD (*n* = 16) groups are shown in Table 1. The body weight of non‐PD group patients was higher than that of PD group patients. Furthermore, the PD group had higher *EGFR* mutations in the tumor. However, there were no significant differences between groups regarding age, sex, carcinoma, clinical stage, PD‐L1 expression, type of ICI used, and lines of ICI therapy.

**TABLE 1:** Demographic of non‐PD and PD groups before ICI administration.

	Non‐PD (*n* = 16)	PD (*n* = 27)	*p*
Age (years)	74 (47–82)	70 (41–84)	0.4892
Sex			
Male	12	17	0.5120
Female	4	10	
Weight (kg)	61.2 (30.7–83.25)	52.2 (38.8–67.2)	0.0366
Histology			
Adenocarcinoma	12	22	0.6928
Squamous cell	3	4	
Other	1	1	
EGFR mutation			
Positive	2	10	0.0328
Negative	12	9	
Not tested	2	8	
Clinical stage			
III	7	7	0.3161
IV	9	20	
PD‐L1 expression			
≥50	3	4	1.0000
<50	10	17	
Not tested	3	6	
ICI treatment			
Nivolumab	4	6	0.9429
Pembrolizumab	8	13	
Atezolizumab	4	8	
Lines of ICI therapy			
First line	2	3	0.6865
Second line	6	7	
≥ Third line	8	17	

*Note*: PD‐L1 expression level was evaluated using tumor proportion score, which was measured using PD‐L1 IHC 22C3 pharmDx, Dako (Agilent Technologies Japan, Ltd., Tokyo, Japan).

Abbreviations: ICI, immune checkpoint inhibitor; PD, progressive disease.

### 3.2 Comparison of the hematological immune biomarkers between the PD and non‐PD groups

There was no significant difference in lymphocyte count between the PD and non‐PD groups before ICI administration and 7–21 and 22–42 days after administration (Table 2). For all patients, blood sampling timing on Days 7–21 were the timing after the first ICI administration. Regarding neutrophil count, there was no significant difference between the PD and non‐PD groups before ICI administration, whereas there was a significant increase at 7–21 days post‐administration in the PD group compared with that in the non‐PD group. At 22–42 days post‐administration, there was no significant difference in neutrophil count between the groups. Lymphocyte counts and neutrophil/lymphocyte ratios did not differ between the two groups before and after ICI administration.

**TABLE 2:** Lymphocyte and neutrophil count in non‐PD and PD groups during 3 months of ICI administration.

	Non‐PD (*n* = 16)	PD (*n* = 27)	*p*
Lymphocyte count (/μL)			
Before ICI administration	1180 (320–2260)	1100 (70–1830)	0.5464
7–21 days after ICI administration	1170 (510–2250)	1130 (473–2760)	0.9544
22–42 days after ICI administration	1175 (248–2350)	1150 (423–2280)	0.9157
Neutrophil count (/μL)			
Before ICI administration	4055 (2340–5560)	4540 (1640–13,270)	0.1483
7–21 days after ICI administration	3559 (1648–5270)	5360 (1235–17,911)	0.0348
22–42 days after ICI administration	3905 (1230–8200)	3920 (2460–24,792)	0.2307
Neutrophil/Lymphocyte ratio			
Before ICI administration	2.941 (1.099–8.967)	3.873 (1.491–150.1)	0.3218
7–21 days after ICI administration	3.165 (1.000–6.050)	4.399 (1.571–31.50)	0.3248
22–42 days after ICI administration	3.460 (0.523–12.27)	3.296 (1.190–48.71)	0.6213

Abbreviations: ICI, immune checkpoint inhibitor; PD, progressive disease.

**FIGURE 2:**
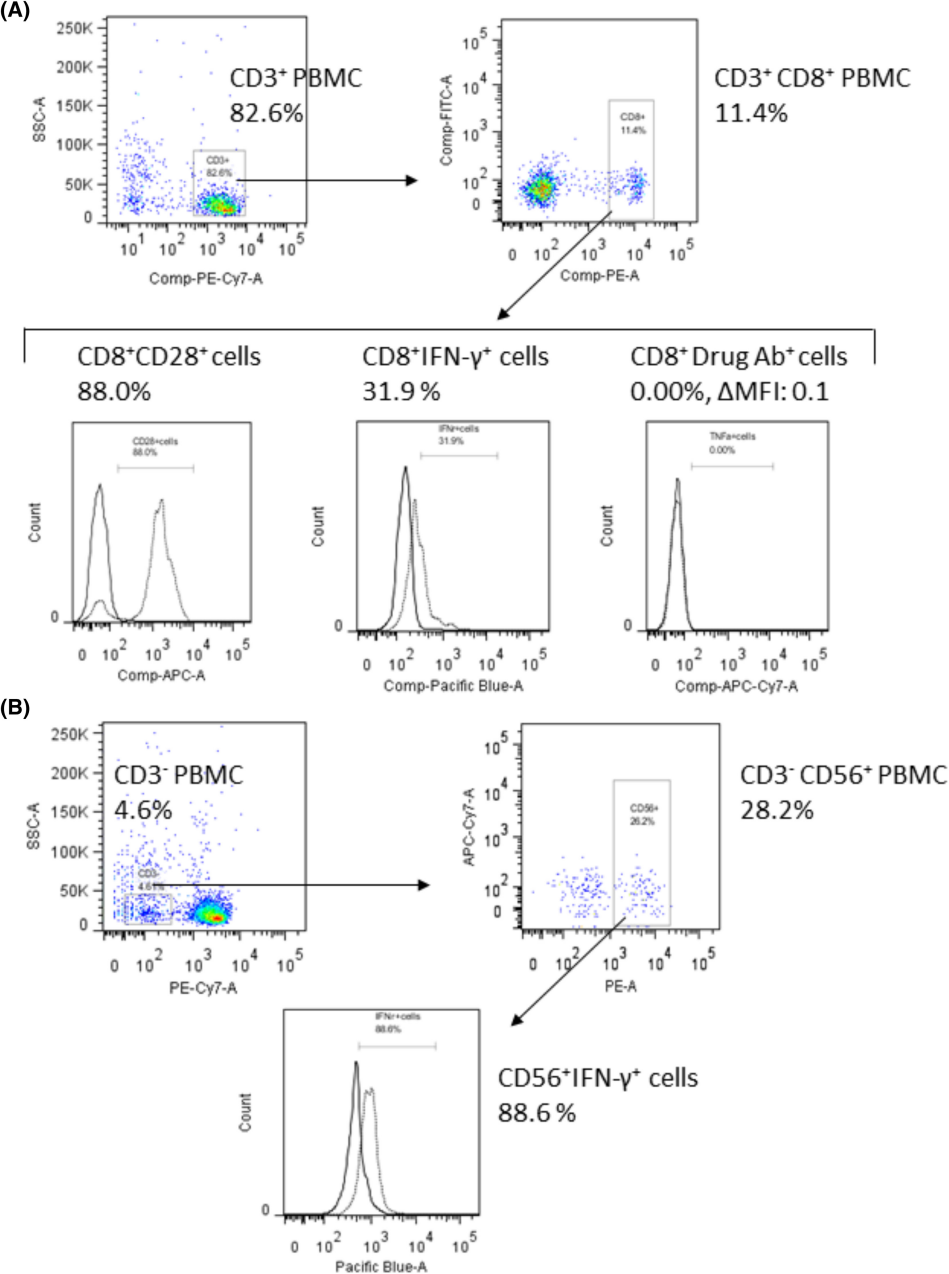
Flow cytometry quantification of the proportion of lymphocyte subsets for CD3^+^ peripheral blood mononuclear cells (A) and CD3^−^ peripheral blood mononuclear cells (B).

Typical flow cytometric quantification of the percentage of lymphocyte subsets is shown in Figure 2. Changes in CD56^+^ cell (NK cell) and CD8^+^ T cell counts in the non‐PD and PD groups at 3 months after ICI administration are shown in Table 3. Concerning the number of CD56^+^ and IFN‐γ^+^ CD56 cells before ICI administration, the PD group had higher counts than the non‐PD group (*p* < 0.0129 and *p* < 0.0422). On the contrary, there was no significant difference between the groups in CD56^+^ cell count at 7–21 and 22–42 days after ICI administration. Regarding CD8^+^ T cell count 7–21 days after ICI administration, the number of IFN‐γ^+^ cells was significantly higher in the non‐PD group than in the PD group (*p* = 0.0478). Similarly, the number of CD28^+^ cells tended to be higher in the non‐PD group than in the PD group (*p* = 0.0722). Furthermore, as shown in Table 4, the number of CD8^+^ CD28^+^ IFN‐γ^+^ T cells 7–21 days after ICI administration and the increase in their count from baseline in the non‐PD group were significantly higher than those in the PD group (*p* = 0.0006 and *p* = 0.0025). Contrarily, there was no significant difference in the CD8^+^ T cell markers between the groups before and 22–42 days after ICI administration. As for ICI binding to CD8^+^ T cells, there was no difference between the groups before and after ICI administration.

**TABLE 3:** Changes in CD56^+^ and CD8^+^ T cell count in non‐PD and PD groups during 3 months of ICI administration.

	Non‐PD (*n* = 16)	PD (*n* = 27)	*p*
CD56^+^ cell count (/μL)			
Before ICI administration	38.3 (9.0–172)	85.3 (2.6–490)	0.0129
7–21 days after ICI administration	71.8 (16.2–331)	116 (14.3–762)	0.1112
22–42 days after ICI administration	110 (12.2–331)	151 (21.2–583)	0.1206
CD56^+^IFN‐γ^+^cell count (/μL)			
Before ICI administration	7.93 (1.5–53.5)	32.6 (0.3–384)	0.0422
7–21 days after ICI administration	18.3 (3.7–100)	32.1 (0.3–292)	0.7190
22–42 days after ICI administration	28.1 (4.7–125)	55.1 (4.1–507)	0.3577
CD8^+^CD28^+^ T cell count (/μL)			
Before ICI administration	103 (10.9–372)	82.5 (1.1–407)	0.3126
7–21 days after ICI administration	118 (60.9–380)	73.8 (20.0–337)	0.0722
22–42 days after ICI administration	97.0 (27.5–416)	94.4 (18.6–380)	0.8105
CD8^+^IFN‐γ^+^T cell count (/μL)			
Before ICI administration	93.3 (7.90–355)	114 (0.3–450)	0.8522
7–21 days after ICI administration	145 (39.3–564)	81.6 (12.8–329)	0.0478
22–42 days after ICI administration	121 (17.8–564)	96.2 (16.6–371)	0.8767
ICI binding to CD8^+^T cell (ΔMFI)			
Before ICI administration	4.3 (0–17.0)	7.2 (0–24.0)	0.4277
7–21 days after ICI administration	0.6 (0–19.0)	3.0 (0–17.0)	0.5025
22–42 days after ICI administration	0.0 (0–29.7)	3.0 (0–28.7)	0.2064

Abbreviations: ICI, immune checkpoint inhibitor; PD, progressive disease.

**TABLE 4:** Changes in CD8^+^CD28^+^IFN‐γ^+^ T cell count in non‐PD and PD groups during 3 months of ICI administration.

	Non‐PD (*n* = 16)	PD (*n* = 27)	*p*
CD8^+^CD28^+^IFN‐γ^+^ T cell count (/μL)			
Before ICI administration	50.4 (2.9–263)	58.6 (0.6–154)	0.7186
7–21 days after ICI administration	72.5 (25.6–286)	31.9 (1.8–136)	0.0006
22–42 days of ICI administration	51.6 (8.5–261)	47.5 (1.8–255)	0.4369
Increased CD8^+^CD28^+^IFN‐γ^+^ T cell count (/μL)			
7–21 days after ICI administration	32.5 (−72.7 to 93.0)	−18.8 (−75.1 to 36.8)	0.0025
22–42 days of ICI administration	8.65 (−83.0 to 109)	−9.25 (−183 to 114)	0.8324

Abbreviations: ICI, immune checkpoint inhibitor; PD, progressive disease.

**FIGURE 3:**
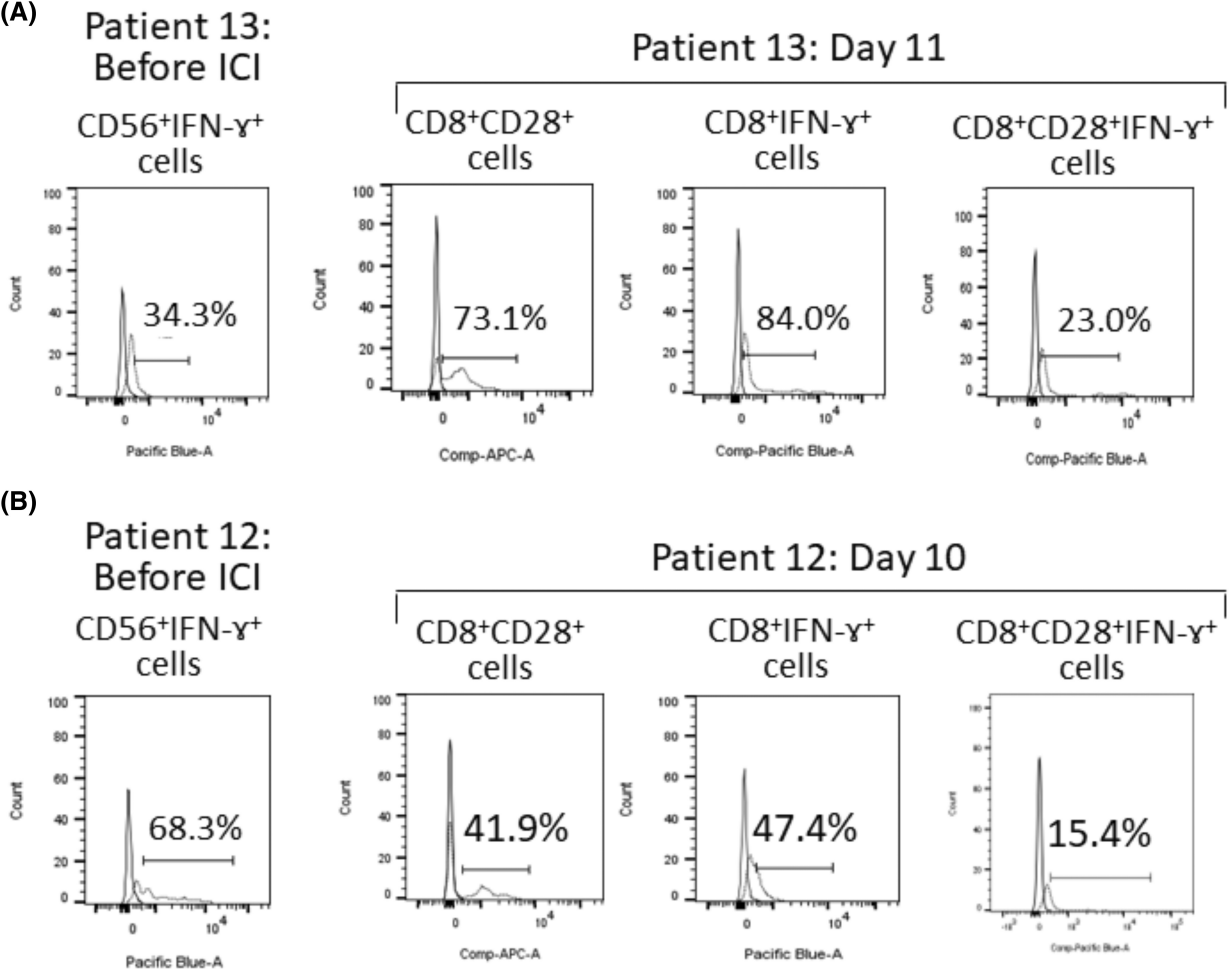
Typical histograms of flow cytometric analysis of CD56^+^IFN‐γ^+^ cells before ICI administration and CD8^+^CD28^+^ cells, CD8^+^IFN‐γ^+^ cells, and CD8^+^CD28^+^IFN‐γ^+^ cells approximately 10 days after ICI administration in a non‐PD patient (Patient 13 day 11) (A) and a PD patient (Patient 12 day 10) (B). Histograms were represented in comparison to the respective isotype control.

In summary, the non‐PD group, which had a better treatment outcome, had lower NK cell activity before ICI administration, and no increase in neutrophil counts and CD8 cell activation with CD28 and IFN‐γ expressions at 7–21 days after ICI administration. Figure 3

### 3.3 ROC curve analysis predicting non‐PD outcome for hematological immune biomarkers

**FIGURE 4:**
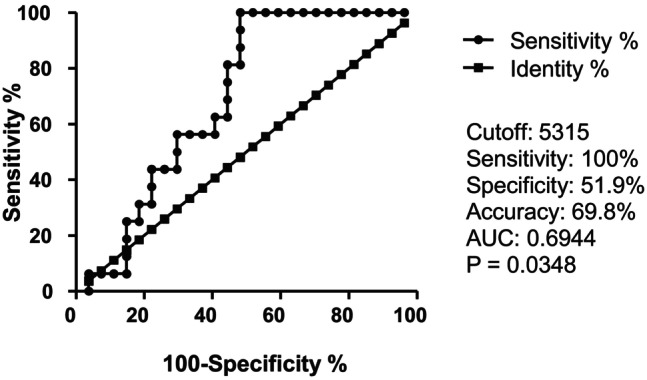
Receiver operator characteristic (ROC) curve analysis of the neutrophil count 7–21 days after ICI administration for predicting non‐PD.

A ROC curve analysis was performed for CD56^+^ and CD8^+^ cell‐related factors. The analysis showed significant differences between the PD and non‐PD groups. Regarding neutrophil count 7–21 days after ICI administration, the cutoff value predicting non‐PD was 5315 cells/μL. The sensitivity, specificity, accuracy, and AUC were 100%, 51.9%, 69.8%, and 0.6944, respectively (*p* = 0.0348) (Figure 4).

**FIGURE 5:**
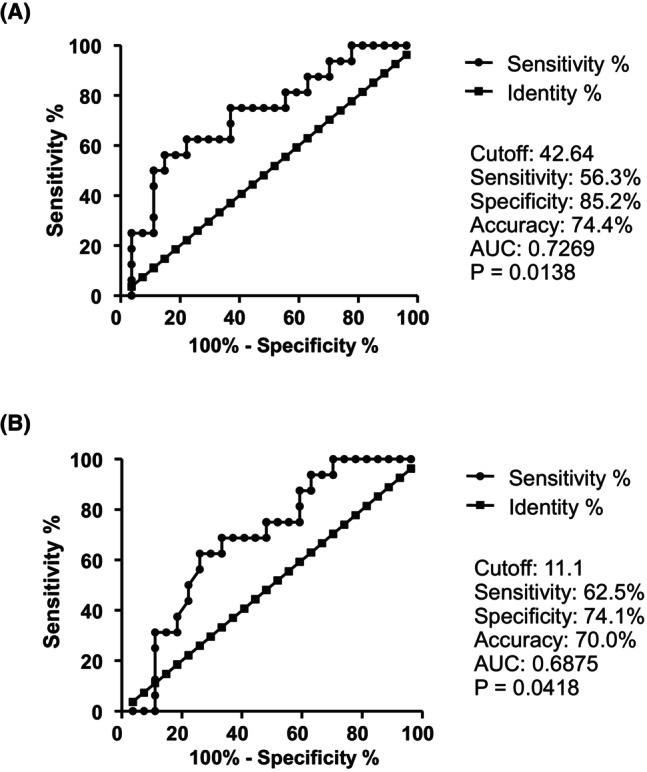
Receiver operator characteristic (ROC) curve analysis of the CD56^+^cell count (A) and CD56^+^IFN‐γ^+^ cell count (B) before ICI administration for predicting non‐PD.

The cutoff value for predicting non‐PD was 42.6 cells/μL for CD56^+^ cell count before ICI administration, with a sensitivity of 56.3%, specificity of 85.2%, accuracy of 74.4%, and AUC of 0.7269 (*p* = 0.0138) (Figure 5A). Similarly, the cutoff value for predicting non‐PD was 11.1 cells/μL for CD56^+^ IFN‐γ^+^ cell count before ICI administration, with a sensitivity of 62.5%, specificity of 74.1%, accuracy of 70.0%, and AUC of 0.6875 (*p* = 0.0418) (Figure 5B).

**FIGURE 6:**
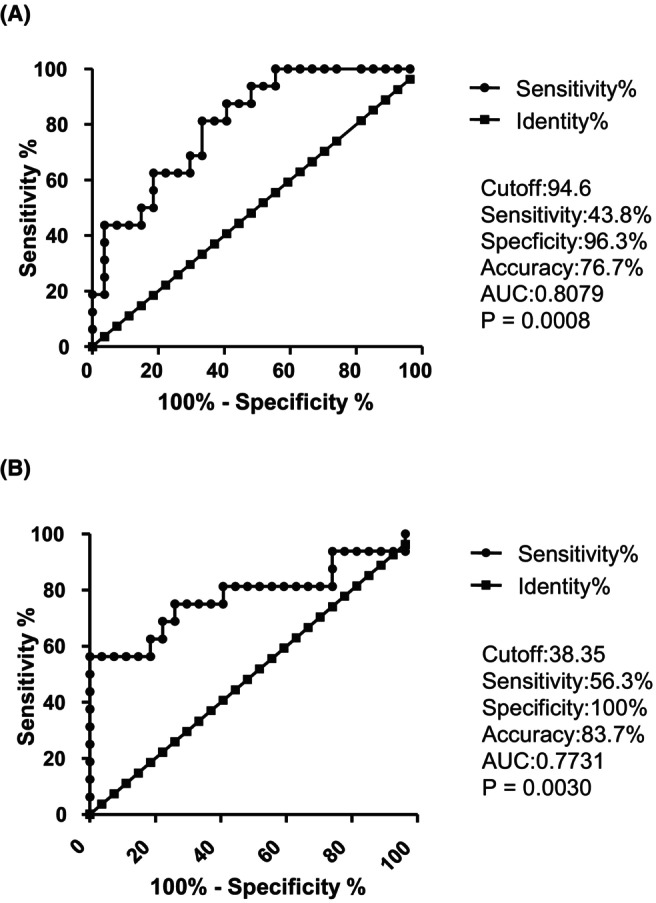
Receiver operator characteristic (ROC) curve analysis of the CD8^+^CD28^+^IFN‐γ^+^ T cell count (A) and increased CD8^+^CD28^+^ IFN‐γ^+^ T cell count (B) 7–21 days after ICI administration for predicting non‐PD.

For CD8^+^ CD28^+^ IFN‐γ^+^ T cell count 7–21 days after ICI administration, the cutoff value predicting non‐progressive disease (PD) was 94.6 cells/μL. The sensitivity, specificity, accuracy, and area under the curve (AUC) were 43.8%, 96.3%, 76.7%, and 0.8079, respectively (*p* = 0.0008). (Figure 6A). Similarly, for increased CD8^+^ CD28^+^ IFN‐γ^+^ T cell count 7–21 days after ICI administration from baseline, the cutoff value predicting non‐PD was 38.4 cells/μL. The sensitivity, specificity, accuracy, and AUC were 56.3%, 100%, 83.7%, and 0.7731, respectively (*p* = 0.0030) (Figure 6B).

In summary, the number of NK cells and activated NK cells before ICI administration had an AUC of around 0.7, which was not sufficient to predict the non‐PD group in clinical practice, while the positivity of CD8^+^ CD28^+^ IFN‐γ^+^ T cell count 7–21 days after ICI administration was highly predictive for it with an AUC of over 0.8.

## 4 DISCUSSION

This study evaluated the relationship between hematological immune biomarkers in the blood and the clinical outcome of non‐PD following ICI administration. The body weight of patients in the non‐PD group was higher than that of patients in the PD group, suggesting a better nutritional status. The non‐PD group also had a lower percentage of *EGFR* mutations in the tumors than the PD group, implying that the tumors tended to be less proliferative. Despite these background differences, the hematological features that facilitated the non‐PD outcome included a low CD56^+^ cell count (CD56^+^ IFN‐γ^+^ cell count) before ICI administration, no increase in neutrophil count, and an increase in the number of activated CD8^+^ cells 7–21 days after ICI administration. To the best of our knowledge, this is the first study to suggest that the CD28^+^ IFN‐γ^+^ T cell count and its increase from baseline may be good indicators of increased activated CD8^+^ cell count and may be associated with good therapeutic outcomes following ICI administration. In particular, CD8^+^ CD28^+^IFN‐γ^+^ T cell count greater than 94.6 cells/μL 7–21 days after ICI administration was highly predictive with an AUC greater than 0.8 in the ROC curve analysis, which may be useful in clinical practice for early prediction of therapeutic response to ICI.

CD28, a co‐stimulatory molecule for T cell activation, is essential for T cells to recognize dendritic cells. Previous studies have shown that CD28 is preferentially dephosphorylated in response to PD‐1 activation by PD‐L1 in an intact cell system and that PD‐1 suppresses T cell function mainly by inactivating CD28 signaling. These findings indicate that co‐stimulatory pathways play key roles in regulating effector T‐cell function and responses to ICI administration.^21^ Moreover, PD‐1 antibodies activate the CD28/B7 pathway to rescue the depletion of CD8^+^ T cells and then achieve anti‐tumor effects.^21^, ^23^ A clinical trial that examined biomarkers predictive of ICI treatment response showed that ICI responders had higher baseline exosomal CD28 expression than non‐responders and that the median progression‐free survival of the high‐CD28‐expressing group was longer than that of the low‐CD28‐expressing group.^24^ In the current study, there was no difference in the number of activated CD8^+^ T cells in the bloodstream before ICI administration between the PD and non‐PD groups, whereas the number of activated CD8^+^ T cells increased in the non‐PD group early after ICI administration (Days 7–21). This phenomenon can be explained by the fact that ICI responders quickly respond to ICIs and that CD8^+^ T cells are activated through increased production of IFN‐γ and CD28. An ex‐vivo functional assay to determine CD8^+^ T cell activation after PD‐1 blockade in circulating peripheral blood showed increased intracellular IFN‐γ^+^ cells.^25^ This study confirmed that the non‐PD group has an increase in CD8^+^ IFN‐γ^+^ cell count early after ICI administration.

Ottonello et al. also showed that in 74 patients with advanced NSCLC treated with nivolumab, patients with longer overall survival had lower NK cell counts at baseline.^26^ This finding supports our result that the non‐PD group had a lower CD56^+^ cell count (CD56^+^ IFN‐γ^+^ cell count) than the PD group before ICI administration. However, a phase II study on pembrolizumab involving patients with advanced NSCLC reported that a higher NK cell count at baseline and the first radiologic evaluation was associated with improved clinical outcomes.^27^ A prospective study of the circulating immune profile in patients with NSCLC receiving nivolumab also showed that at baseline, the clinical benefit group (*n* = 19) had a higher number of active NK and PD‐1^+^CD8^+^ cells than non‐responders (*n* = 12).^28^ Moreover, a prospective exploratory study of 40 patients with small‐cell lung cancer treated with chemotherapy and ICIs showed that disease progression correlated with a low NK cell count.^29^ These results indicate that the specific function of NK cells in immunotherapy and their potential as biomarkers for predicting response to ICIs need further investigation.

An elevated neutrophil/lymphocyte ratio indicates chronic inflammation and reflects the immune status of patients with different malignancies.^30^ Several studies have investigated the negative prognostic value of a high neutrophil/lymphocyte ratio in patients with NSCLC receiving immunotherapy. In addition, an analysis of clinical blood test data from 249 patients with NSCLC treated with ICIs revealed that a high neutrophil/lymphocyte ratio of 5 before and after ICI administration was associated with a low survival rate.^31^ Suh et al. also reported that a neutrophil/lymphocyte ratio ≥5 at 6 weeks post‐ICI administration is associated with poor progression‐free survival and overall survival.^32^ As mentioned earlier, a low neutrophil count (<4000/μL) is reportedly associated with lower response rates in ICI treatment.^17^ Taken together, these reports support our results showing that no increase in neutrophil count 7–21 days after ICI administration is associated with a good clinical outcome, although our data did not show a difference of the neutrophil/lymphocyte ratio between the non‐PD and PD groups. Therefore, in addition to a neutrophil/lymphocyte ratio, an absence of an increase in neutrophil count after ICI administration could be a good predictor of the therapeutic effect of ICIs.

This study has some limitations. First, the findings of this pilot study are limited by the small number of patients enrolled. Second, this study focused on the early phase clinical outcome (initial 3 months) after ICI administration and did not perform a multivariate analysis of factors associated with non‐PD because of the small sample size. However, this exploratory prospective case–control study provides solid clinical evidence with a large statistical difference in predicting response to ICIs, especially concerning the dynamics of CD8^+^CD28^+^IFN‐γ^+^ T cells after ICI administration.

To conclude, this exploratory study showed a relationship between peripheral blood immune cell reactivity and clinical response to ICIs in patients with NSCLC. We found that the lack of an increase in the number of neutrophils and the increase in circulating CD8^+^CD28^+^IFN‐γ^+^ T cells after ICI administration were associated with favorable clinical outcomes. The findings will contribute to selecting patients with a favorable therapeutic response and early prediction of therapeutic response to ICI and warrant further validation in larger studies in the future. These findings will be of interest to healthcare professionals involved in cancer treatment, immunotherapy, and personalized drug therapy.

## AUTHOR CONTRIBUTIONS

**Takuya Iwamoto:** Conceptualization (lead); data curation (lead); formal analysis (lead); funding acquisition (lead); investigation (lead); methodology (lead); project administration (lead); resources (lead); validation (lead); writing – original draft (lead). **Hajime Fujimoto:** Conceptualization (supporting); investigation (supporting); writing – review and editing (equal). **Tetsu Kobayashi:** Conceptualization (supporting); investigation (supporting); supervision (supporting); writing – review and editing (supporting). **Esteban C. Gabazza:** Validation (equal); writing – review and editing (supporting).

## CONFLICT OF INTEREST STATEMENT

The authors declare that they have no conflicts of interest regarding the content of this article.

## ETHICS STATEMENT

Approval of the research protocol by an Institutional Review Board: Yes. Informed Consent: Yes. Written informed consent was obtained from each participant.

## Data Availability

The raw data used to support the findings of this study are available from the corresponding author upon request.
